# Efficacy and safety of sacubitril valsartan in treating heart failure with midrange ejection fraction after acute myocardial infarction in diabetic patients

**DOI:** 10.1097/MD.0000000000028729

**Published:** 2022-02-04

**Authors:** Fanhao Ye, Hebo Li, Xiaoshu Chen, Yi Wang, Wei Lin, Hao Chen, Shiwei Huang, Sisi Han, Fanlu Guan, Zhouqing Huang

**Affiliations:** a Department of Cardiology, Wenzhou People's Hospital, The Third Affiliated Hospital of Shanghai University, Wenzhou, Zhejiang, China; b Wenzhou Medical University, Wenzhou, Zhejiang, China; c Department of Cardiology, The First Affiliated Hospital of Wenzhou Medical University, Wenzhou, Zhejiang, China.

**Keywords:** acute myocardial infarction, diabetes mellitus, heart failure with midrange ejection fraction, sacubitril valsartan

## Abstract

Objective to evaluate the clinical efficacy and safety of sacubitril valsartan in the treatment of heart failure (HF) with midrange ejection fraction after acute myocardial infarction (AMI) in diabetic patients. From January 2015 to July 2020, HF patients with diabetes mellitus complicated with AMI were retrospectively analyzed. According to the medication, they were divided into 2 groups, that is, sacubitril valsartan group (84 cases) and valsartan group (86 cases). Valsartan group took valsartan capsule (80 mg/capsule, Beijing Novartis Pharmaceutical Co., Ltd) 80 mg, qd, on the basis of routine treatment. On the basis of routine treatment, the sacubitril valsartan group took sacubitril valsartan sodium tablets (50 mg/tablet, Beijing Novartis Pharmaceutical Co., Ltd), the initial dose was 25 mg, bid, and gradually increased to the target dose according to the patient's blood pressure. After 12 months of treatment, the independent sample *t* test showed that the left ventricular end diastolic dimension in the sacubitril valsartan group was lower than that in the valsartan group [(47.26 ± 4.71) mm vs (50.05 ± 5.62) mm, *P* < .001]. The left ventricular ejection fraction in the sacubitril valsartan group was higher than that in the valsartan group [(54.76 ± 4.24)% vs (49.28 ± 3.74)%, *P* < .001]. χ^2^ inspection showed that the readmission rate in the sacubitril valsartan group was lower than that in the valsartan group (7.14% vs 18.60%, *P* < .05). Sacubitril valsartan has good safety and tolerability in patients with diabetes mellitus complicated with AMI who have HF with midrange ejection fraction. Compared with valsartan, sacubitril valsartan can improve the left ventricular function better and reduce the readmission rate due to HF in these patients.

## 1 Introduction

Diabetes mellitus (DM) is considered as an important risk factor for heart failure (HF), death and re infarction in patients with acute myocardial infarction (AMI).^[1]^ The incidence of short and long term HF in diabetic patients after AMI is 2∼3 times of nondiabetic patients.^[2,3]^ HF is a common adverse cardiac event after AMI, which can significantly increase the risk of death. A retrospective cohort study found that. the incidence of HF in patients with acute non-ST segment elevation myocardial infarction and ST segment elevation myocardial infarction during hospitalization is 13.6% and 14.8%, respectively, and the incidence of HF within 1 year can reach 23.4% and 25.4%.^[4]^

Recently, there have been many studies on the treatment of HF after AMI with sacubitril valsartan. However, since the exact pathogenesis of HF after AMI is more complicated, whether sacubitril valsartan can treat diabetic patients with heart failure with midrange ejection fraction (HFmrEF) after AMI has no consistent conclusion. The prevalence of HFmrEF has increased significantly, but its treatment is still a challenge for clinicians. HFmrEF is a hemodynamic state. The heart can not meet the circulatory needs of the body, or at the cost of increasing left ventricular filling pressure. There is strong but indirect evidence that hypertension treatment can effectively prevent HFmrEF, but the data on improving the survival benefits of patients with HFmrEF are not clear. Active blood pressure control is associated with a lower risk of hospitalization for HF. At present, there is little evidence that renin angiotensin aldosterone system (RAAS) inhibition is beneficial to HFmrEF, but the data show that it may benefit those with ejection fraction <55%.^[5]^

This article aims to compare the changes of related indexes and the incidence of main adverse reactions in DM patients with HFmrEF after AMI treated by sacubitril valsartan and valsartan, and to explore the efficacy and safety of sacubitril valsartan in treating DM patients with HFmrEF after AMI.

## 2 Materials and methods

### 2.1 Patients

A total of 170 patients with DM and AMI treated by percutaneous coronary intervention were collected from Department of Cardiology, Wenzhou people's Hospital from January 2015 to July 2020, including 120 males and 50 females, aged (62.89 ± 12.2) years. According to the difference of treatment regimen, they were divided into 2 groups, namely, the 84 cases of sacubitril valsartan group and 86 cases of valsartan group. Inclusion criteria: meet the diagnostic criteria of AMI: chest pain lasts more than 30 minutes and sublingual nitroglycerin can not be relieved ST segment elevation of 2 adjacent leads >0.1 mv or ST segment down >0.2 mv; the content of creatine kinase isozyme or troponin I increased at least 3 times. Meet the criteria for chronic HF: patients diagnosed with HF according to the diagnostic criteria of Chinese guidelines for the diagnosis and treatment of HF 2014.^[6]^ The left ventricular ejection fraction (LVEF) ranged from 40% to 50%. The diagnosis of diabetes accords with the diagnostic criteria of WHO diabetes in 1999: diabetes symptoms and random blood glucose equal to 11.1 mmol/L or fasting blood glucose equal to 7.0 mmol/L or oral glucose tolerance test 2 hours blood glucose is more than 11.1 mmol/L. Exclusion criteria included: patients allergic to sacubitril valsartan or valsartan; patients with cardiac insufficiency caused by other heart diseases (such as dilated cardiomyopathy, valvular heart disease, hypertensive cardiac insufficiency, etc); acute decompensation of chronic cardiac insufficiency; blood potassium was greater than 5.5 mmol/L; severe renal insufficiency; the serum potassium was more than 5.4 mmol/L. This study met the medical ethics standards and was approved after discussion by the hospital ethics committee.

### 2.2 Grouping and treatment

According to the difference of treatment regimen, they were divided into 2 groups, that is, 84 cases in sacubitril valsartan group and 86 cases in valsartan group. All patients were given conventional medication after DM and AMI, while the trial group was given sacubitril valsartan (Beijing Novartis Pharma Ltd, 50 mg/starting dose 25 mg, bid, gradually increasing to the target dose according to the blood pressure of the patients), and the control group was given valsartan (80 mg, qd, Beijing Novartis Pharma Ltd, 50 mg).

### 2.3 Data collection

The mean systolic blood pressure, N-terminal pro-brain natriuretic peptide, serum creatinine and low density lipoprotein-cholestero, hemoglobin A1c, fasting blood glucose, and cardiac function rating (Killip classification) within 24 hours after percutaneous coronary intervention were collected.

The levels of LVEF and the left ventricular end diastolic dimension (LVDD) at admission and 12 months after treatment were measured by transthoracic echocardiography.

The number of readmissions due to HF within 12 months was counted.

### 2.4 Statistical methods

SPSS version 23.0 for Windows (IBM Corp.; Armonk, NY) was used for statistical analysis. The categorical variables were represented as frequencies and percentages, and the continuous variables were reported as mean ± standard deviation. The categorical variables were compared using the chi-squared test or Fisher exact test. The continuous variables were compared using the Student *t* test or Mann–Whitney *U* test, based on the distribution of the data. A *P*-value of <.05 was considered statistically significant.

## 3 Results

### 3.1 Patient baseline characteristics

There was no significant difference in baseline data such as age, gender, hypertension, systolic blood pressure, serum creatinine, severity of coronary artery, cardiac function rating (Killip classification), preoperative LVDD, preoperative LVEF, and preoperative N-terminal pro-brain natriuretic peptide between sacubitril valsartan group and valsartan group (all *P* > .05), as shown in Table 1.

**Table 1:** Comparison of data between sacubitril valsartan group and valsartan group.

Characteristic	Salkubatroxartan group (84)	Valsartan group (86)	*t* or χ^2^	*P*
Age (yr), mean ± SD	62.29 ± 12.82	63.49 ± 11.61	−0.642	.522
Gender (male), n (%)	52 (61.90%)	56 (65.12%)	0.664	.750
Hypertension, n (%)	56 (66.67%)	52 (60.47%)	0.705	.429
SP (mm Hg) mean ± SD	124.90 ± 18.68	128.91 ± 16.52	−1.481	.141
Severity of myocardial infarction, n (%)				
Single vessel lesion	7 (8.33%)	11 (12.79%)	0.892	.456
Double vessel lesion	20 (23.81%)	19 (22.62%)	0.071	.856
Three vessel lesion	56 (66.67%)	56 (65.12%)	0.045	.872
HbA1c (%), mean ± SD	8.40 ± 1.67	8.05 ± 1.22	1.567	.119
FBG (mmol/L), mean ± SD	10.41 ± 3.69	9.91 ± 3.54	0.902	.368
NT-proBNP (ng/L), mean ± SD	679.10 ± 623.32	666.14 ± 407.78	0.161	.872
SCr (umol/L), mean ± SD	69.69 ± 21.52	75.05 ± 25.77	−1.468	.144
LDL-C (mmol/L), mean ± SD	2.43 ± 0.83	2.54 ± 0.79	−0.825	.410
Killip classification, n (%)				
I	22 (26.19%)	24 (27.91%)	0.801	.864
II	26 (30.95%)	26 (30.23%)	0.919	1.000
III	14 (16.67%)	16 (18.60%)	0.740	.841
IV	22 (26.19%)	20 (23.26%)	0.657	.723
Drug use during treatment, n (%)				
β-blocker	76 (90.48%)	74 (86.05%)	0.370	.477
Spironolactone	26 (30.95%)	30 (34.88%)	0.586	.627
Digitalis	32 (38.10%)	30 (34.88%)	0.664	.750
Diuretic	2 (2.38%)	2 (2.33%)	0.981	1.000
LVEF (%), mean ± SD	44.69 ± 4.60	44.65 ± 4.42	0.057	.955
LVDD (mm), mean ± SD	51.52 ± 6.20	50.05 ± 5.62	1.552	.123

FBG = fasting blood glucose, HbA1c = hemoglobin A1c, LDL-C = low density lipoprotein-cholestero, LVDD = left ventricular end diastolic dimension, LVEF = left ventricular ejection fraction, NT-proBNP = N-terminal pro-brain natriuretic peptide, SCr = serum creatinine, SP = systolic pressure.

### 3.2 Comparison of left ventricular function

After 12 months of treatment, LVDD of sacubitril valsartan group was lower than that of valsartan group, and LVEF of sacubitril valsartan group was higher than that of valsartan group, as shown in Table 2.

**Table 2:** Comparison of left ventricular function and rehospitalization rate between sacubitril valsartan group and valsartan group after 12 months of treatment.

Characteristic	Salkubatroxartan group (84)	Valsartan group (86)	*t* orχ^2^	*P*
LVEF (%), mean ± SD	54.76 ± 4.24	49.28 ± 3.47	9.235	<.001
LVDD (mm), mean ± SD	47.26 ± 4.71	50.12 ± 5.62	−3.643	<.001
Readmission rate, n (%)	6 (7.14%)	16 (18.60%)	0.026	.038

LVDD = left ventricular end diastolic dimension, LVEF = left ventricular ejection fraction.

### 3.3 Comparison of main adverse reactions

During the 12-month follow-up, there was no discontinuation of drugs due to hypotension in the sacubitril valsartan group and valsartan group. There were no patients who stopped the drug due to severe renal insufficiency and hyperkalemia in both groups. No patient developed angioedema. The readmission rate due to HF in the sacubitril valsartan group was lower than that in the control group, as shown in Table 2.

## 4 Discussion

The incidence of HF after AMI is related to ventricular remodeling, neuroendocrine system activation and inflammatory factors.^[7]^ Sacubitril valsartan can be decomposed into enkephalinase inhibitors under the action of liver enzymes. Enkephalinase is an endonuclease that can hydrolyze a variety of endogenous vasoactive peptides, such as natriuretic peptide, angiotensin, bradykinin and other peptides, including vasodilators such as natriuretic peptide and bradykinin, as well as vasoconstrictors such as angiotensin. Enkephalinase is the key enzyme for the degradation of natriuretic peptide. After enkephalinase is inhibited, the degradation of natriuretic peptide decreases correspondingly, but at the same time, the level of vasoconstrictors such as angiotensin will also increase. Due to the mutual offset of vasodilation and vasoconstriction, the effect of treating HF with enkephalinase inhibitor alone may be poor.^[8]^ In the follow-up study, the combination of enkephalinase inhibitor and angiotensin receptor antagonist was used to prepare salkubatron valsartan. Sacubitril valsartan plays a role in protecting natriuretic peptide system. Valsartan is an angiotensin II-1 receptor blocker, which can inhibit the role of angiotensin. The 2 cooperate to make up for the disadvantages of simple counteraction of vasodilator and vasoconstrictor effects of sacubitril valsartan. Through the dual effects of inhibiting RAAS and protecting natriuretic peptide system, sacubitril valsartan improves the balance of neurohormones, plays the role of natriuretic diuresis, dilating blood vessels, improving the level of cyclic guanosine phosphate, reducing cardiac preload and preload, inhibiting myocardial hypertrophy and ventricular remodeling in the treatment of HF.^[9,10]^

An animal model study on AMI and HF showed that sacubitril valsartan significantly reduced myocardial infarction area than valsartan, and sacubitril valsartan significantly reduced cTnI level in the acute stage of myocardial infarction. Sacubitril valsartan improves left ventricular function caused by ischemic cardiomyopathy and reduces scar area after myocardial infarction. Insulin resistance and hyperinsulinemia in diabetes patients can activate SNS and RAAS, promote oxidative stress and mitochondrial dysfunction, lead to myocardial hypertrophy and fibrosis, and coronary microcirculation disturbance, eventually leading to HF.^[11]^ PARADIGM-HF studies show that sacubitril valsartan may improve insulin resistance in diabetic patients through regulating the endocrine system, and has a positive effect on patients with HF and DM. This study showed that sacubitril valsartan significantly improved the left ventricular function in patients with HFmrEF caused by ischemic cardiomyopathy. PARADIGM-HF through comparing the efficacy of sacubitril valsartan and enalapril in the treatment of HFmrEF patients, it was found that sacubitril valsartan can reduce the risk of cardiovascular death by 20%, the risk of rehospitalization for HF by 21% and the risk of all-cause death by 16%.^[12]^ This study found that compared with valsartan patients, sacubitril valsartan could improve heart function in patients with DM complicated with AMI and reduce the readmission rate.

This study shows that the LVDD in the sacubitril valsartan group is better than that in the valsartan group, which shows that the effect of sacubitril valsartan on improving ventricular remodeling is not limited to ejection fraction reducing HF, but also significant in ejection fraction preserving HF. In addition, the effect of sacubitril valsartan is better than valsartan in improving mind remodeling. This is due to the strong inhibition of enkephalinase by sacubitril through lbq657 (the active metabolite of the prodrug sacubitril).

This study showed that there were no adverse reactions such as drug-induced renal insufficiency and hyperkalemia in both sacubitril valsartann group and valsartan group. This shows that the safety of sacubitril valsartan in this study is equivalent to that of valsartan in patients with normal renal function. In this study, no hypotension with obvious symptoms or angioedema occurred in both groups. There is a small sample size limit in this study, so the incidence of the above common adverse reactions is 0%. PARADIGM-HF study has confirmed that the incidence of adverse reactions such as hyperkalemia or abnormal renal function in sacubitril valsartan group is lower than that in enalapril group, and has good safety and tolerance. Therefore, sacubitril valsartan is relatively safe in patients with DM complicated with AMI who has HFmrEF.

To sum up, compared with valsartan, sacubitril valsartan can improve left ventricular function in diabetics complicated with AMI with HFmrEF, and reduce the readmission rate due to HF. It is a new way to prevent and treat HF after AMI in diabetic patients. The advantage of this study is to take the lead in the study of diabetic patients with HFmrEF after AMI, and the results show that sacubitril valsartan has a significant benefit in such patients, supplemented by the evidence of the use of sarkovate valsartan in patients with HF. However, the study sample is small and the follow-up period is short. Therefore, more trials are needed, and long time studies are needed to evaluate the clinical efficacy of reducing the chronic HF after AMI in diabetic patients.

## Acknowledgments

The authors thank the patients for participating and supporting this study.

## Author contributions

FY, HL, and ZH designed the study. FY, HL, and ZH drafted the manuscript. FY, HL, XC, YW, HC acquired, analyzed, and interpreted the data. FY, WL, HL, SH, and FG edited the manuscript. All authors read and approved the final manuscript.

**Conceptualization:** Fanhao Ye, Zhouqing Huang.

**Data curation:** Fanhao Ye, Hebo Li.

**Formal analysis:** Fanhao Ye, Hebo Li, Wei Lin.

**Investigation:** Fanhao Ye, Shiwei Huang.

**Methodology:** Fanhao Ye, Zhouqing Huang.

**Project administration:** Fanhao Ye, Wei Lin, Zhouqing Huang.

**Resources:** Fanhao Ye, Fanlu Guan.

**Software:** Fanhao Ye, Xiaoshu Chen.

**Supervision:** Fanhao Ye, Hao Chen, Zhouqing Huang.

**Validation:** Fanhao Ye, Zhouqing Huang.

**Visualization:** Fanhao Ye, Xiaoshu Chen, Yi Wang, Sisi Han, Zhouqing Huang.

**Writing – original draft:** Fanhao Ye, Hebo Li, Zhouqing Huang.

**Writing – review & editing:** Fanhao Ye, Hebo Li, Zhouqing Huang.
